# Characterization of the lncRNA-mediated ceRNA regulatory networks in preeclampsia by integrated bioinformatics

**DOI:** 10.1038/s41598-023-44059-w

**Published:** 2023-10-12

**Authors:** Liping Zhu, Chengfeng Liu, Yongmei Xu, Yongfei Yue, Jianying Tao, Chunhua Zhang, Xing Zhang, Xinfang Zhou, Ye Song

**Affiliations:** 1 Department of Obstetrics, The Affiliated Suzhou Hospital of Nanjing Medical University, Suzhou Municipal Hospital, Gusu School, Nanjing Medical University, No. 26 Daoqian Street, Gusu District, Suzhou, 215000 Jiangsu China; 2 School of Chemistry and Life Science, Suzhou University of Science and Technology, Suzhou, 215009 China

**Keywords:** Biochemistry, Molecular biology, Diseases

## Abstract

Preeclampsia (PE) is a significant threat to all pregnancies that is highly associated with maternal mortality and developmental disorders in infants. However, the etiopathogenesis of this condition remains unclear. This study aims to explore the regulatory roles of long noncoding RNAs (lncRNAs) and the mediated competing endogenous RNAs (ceRNA) in the etiopathogenesis of PE through analysis of lncRNA expression patterns in PE and healthy pregnant women (HPW), as well as the construction of lncRNA-mediated ceRNA regulatory networks using bioinformatics. A total of 896 significant differentially expressed lncRNAs, including 586 upregulated lncRNAs and 310 downregulated lncRNAs, were identified in comparison between PE and HPW. Analysis of these differential expressed lncRNAs revealed their predominant enrichment in molecular functions such as sphingosine-1-phosphate phosphatase activity, lipid phosphatase activity, phosphatidate phosphatase activity, thymidylate kinase activity, and UMP kinase activity. Moreover, these differential expressed lncRNAs were predominantly enriched in KEGG analyses such as fat digestion and absorption, lysine degradation, ether lipid metabolism, glycerolipid metabolism, and sphingolipid metabolism. Two ceRNA regulatory networks were constructed based on ceRNA score, including one that had 31 upregulated lncRNAs, 11 downregulated miRNAs, and 34 upregulated mRNAs, while the other contained 128 downregulated lncRNAs, 40 upregulated miRNAs, and 113 downregulated mRNAs. These results may provide a clue to explore the roles of lncRNAs in the etiopathogenesis of PE.

## Introduction

Preeclampsia (PE) is a serious condition that typically develops after 20 weeks of pregnancy^1^ and is characterized by high blood pressure and proteinuria^2^. It is a leading cause of maternal mortality^3^ and can also cause brain developmental disorders^4^, including intellectual disability, in infants, resulting in a significant burden on healthcare services^5^.

While the etiology of PE remains unclear, research has increasingly focused on several potential causes, including immunological intolerance^6,7^ and angiogenesis imbalance^5^, inflammatory cytokine disorders^8^ and immune maladjustment^9^. In addition, multi-omics approaches have identified potential metabolic disruptions in steroid hormones and imbalanced metabolism of androgens and estrogens in Placenta^10,11^ and a few lncRNA-mediated ceRNA regulatory mechanisms^12^, such as LncRNA-XIST regulation of KCNJ16 via miR-340-5p sponge^13^, CircFN1 modulation of ATF2 via miR-19a/b-3p sponge^14^, circ_0008726 regulation of RYBP via miR-345-3p sponge^15^, and Circ_0015382 acting as a miR-149-5p sponge to modulate the expression of TFPI2, associated with the onset and development of PE^16^. Increasing data for the etiopathogenesis of PE was from the cross-talk among circRNA, miRNA, and mRNAs. Recently, increasing reports demonstrated that dysregulated lnRNAs identified from trophoblast cells and placentas were predicted to be closely related to the pathogenesis of PE^17–20^. However, there is still only several reported data from the lncRNA-mediated ceRNA to describe the etiopathogenesis of PE from the patients using their peripheral blood^19–25^. In addition, biochemical tests of the PE showed that aminotransferases, creatinine, total bilirubin and Urea levels in were increased in the serum from pregnant women, and creatinine level in serum was predicted as the best diagnostic marker for PE^26^. A deleterious effect on renal and liver function happened in the PE followed by the changes of biochemical indexes^26^.

This study aimed to investigate the demographic characteristics, infant outcomes, serum biochemical indices, and expression profiles of lncRNAs in PE and build integrated bioinformatics-based lncRNA-mediated ceRNA regulatory networks to better understand the etiopathogenesis of PE.

## Materials and methods

### PE patients

Pregnant women with pre-eclampsia (PE) and healthy pregnant controls (HPW) were recruited in this study, with three participants in each group identified from the Maternity and Child Health Care Affiliated Hospital of Jiaxing University and the basic characteristics of the included subjects were shown in our previous report^27^. From the 30 pregnant women diagnosed with pre-eclampsia based on gestational hypertension and proteinuria and reported clinical features of PE, the demographic characteristics, infant outcomes, and serum biochemical indices, including glutamic-pyruvic transaminase (ALT) and glutamic oxalacetic transaminase (AST), were investigated. Ethics approval was obtained from the Ethics Committee of the Faculty of Medicine, and written informed consent was obtained from all participants. The study was approved by the research ethics committee for clinical studies at Suzhou Municipal Hospital, Jiangsu, China and all experiments were performed in accordance with relevant guidelines and regulations.

### Total RNAs extraction

Total RNA was extracted from the fasting whole blood sample (3.0 mL) of each participant—the PE group and HPW group—with a Blood Cell Lysis Buffer kit (Tiangen, China). The purified leukocyte was used for extraction of total RNA with TRIzol reagent (Invitrogen, Carlsbad, USA) using standard protocols.

### rRNA-depleted and RNA sequencing

The high-quality purified RNA was subjected to treatment with the TruSeq Stranded Total RNA with Ribo-Zero Gold kit (Illumina, RS-122-2301) to remove ribosomal RNA. The first strand cDNA was synthesized with the SuperScript II Reverse Transcriptase kit, followed by second strand cDNA synthesis with Second Strand Marking Master Mix. Purification of cDNA was performed using AMPure XP beads. The adenylated 3′ ends were then added with A-tailed Mix, and adapters were ligated with RNA Adapter Index. AMPure XP Beads were then used for further purification of the cDNAs, adenylated 3′ ends, and adapters and finally, to purify the DNA fragments. We built cDNA libraries with the TruSeq Stranded Total RNA with Ribo-Zero Gold kit; the HiSeqTM4000 sequencing platform was used to sequence the six cDNA libraries by Shanghai OE Biotech, Shanghai, China, and the sequencing reads are accessible in PRJNA665923.

### Bioinformatics analysis

High-throughput sequencing reads were processed with Trimmomatic software^28^ to remove adapters. The high-quality clean reads were collected by removing low-quality data. Hisat2^29^ was used to map clean reads to the reference sequence produced by the human reference genome (GRCh38.p12). The candidate long non-coding RNA (lncRNA) transcripts were selected to compare with the reference sequence using Cuffcompare software^30^. These transcripts with coding potential were screened out by using Coding Potential Calculator (CPC)^31^, Coding-Non-Coding Index (CNCI)^32^, Pfam^33^, and PLEK^34^ to obtain predicted lncRNA sequences. The counts were normalized using the DESeq (2012) R package, and the difference between the two groups was calculated using p-value and fold change values with the nbinomTest function. LncRNAs with p-values ≤ 0.05 and a fold change ≥ 2 were designated as differentially expressed. Geno ontology (GO) and Kyoto encyclopedia of genes and genomes (KEGG) enrichment were conducted for the potential roles of these dysregulated lncRNAs. For the correction of p-values, the false discovery rate was calculated with the Benjamini and Hochberg multiple tests. LncRNA-mediated ceRNA networks were built using differentially expressed miRNAs and mRNAs with the ceRNA_score.

### Statistical analysis

The statistical software GraphPad Prism for Windows version 5.0 (GraphPad Software, San Diego, CA, USA) was used to plot all graphs, and the student-T test was used for statistical analysis. Statistical significance was indicated with **** p < 0.0001 and * p < 0.05.

## Results

### Demographic characteristics and infant outcomes

**Figure 1:**
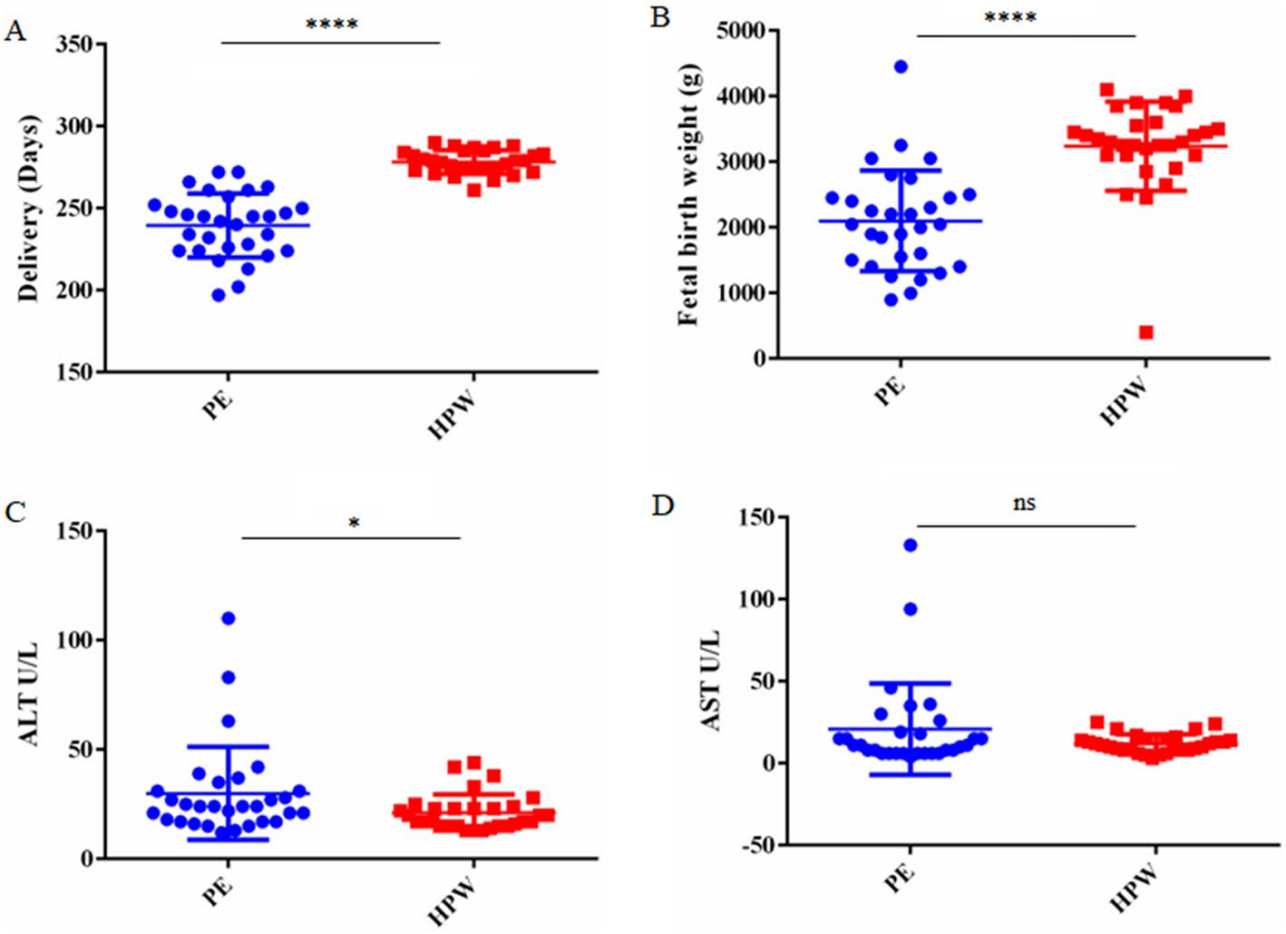
Demographic characteristics of PE patients and fetal birth weight. (**A**) Delivery pregnancy week between the PE group and the control HPW group. (**B**) Fetal birth weight of PE group and control HPW group. The level of ALT (**C**) and AST (**D**) in serum from PE group and control HPW group.

A total of thirty patients with gestational hypertension and preeclampsia were strictly selected, and healthy pregnant women (HPW) were recruited as controls from hospitals. The age range of participants was 20–40 years. The delivery pregnancy week in the preeclampsia group was significantly lower than that of the control group (Fig. 1A). The fetal birth weight of the preeclampsia group was significantly lower than the control group (Fig. 1B). Compared to the control group, the levels of ALT and AST were significantly higher in the serum of the preeclampsia group (Fig. 1C, D). These results suggest that preeclampsia leads to infant developmental disorders and liver injury.

### Potential lncRNAs exploration

**Figure 2:**
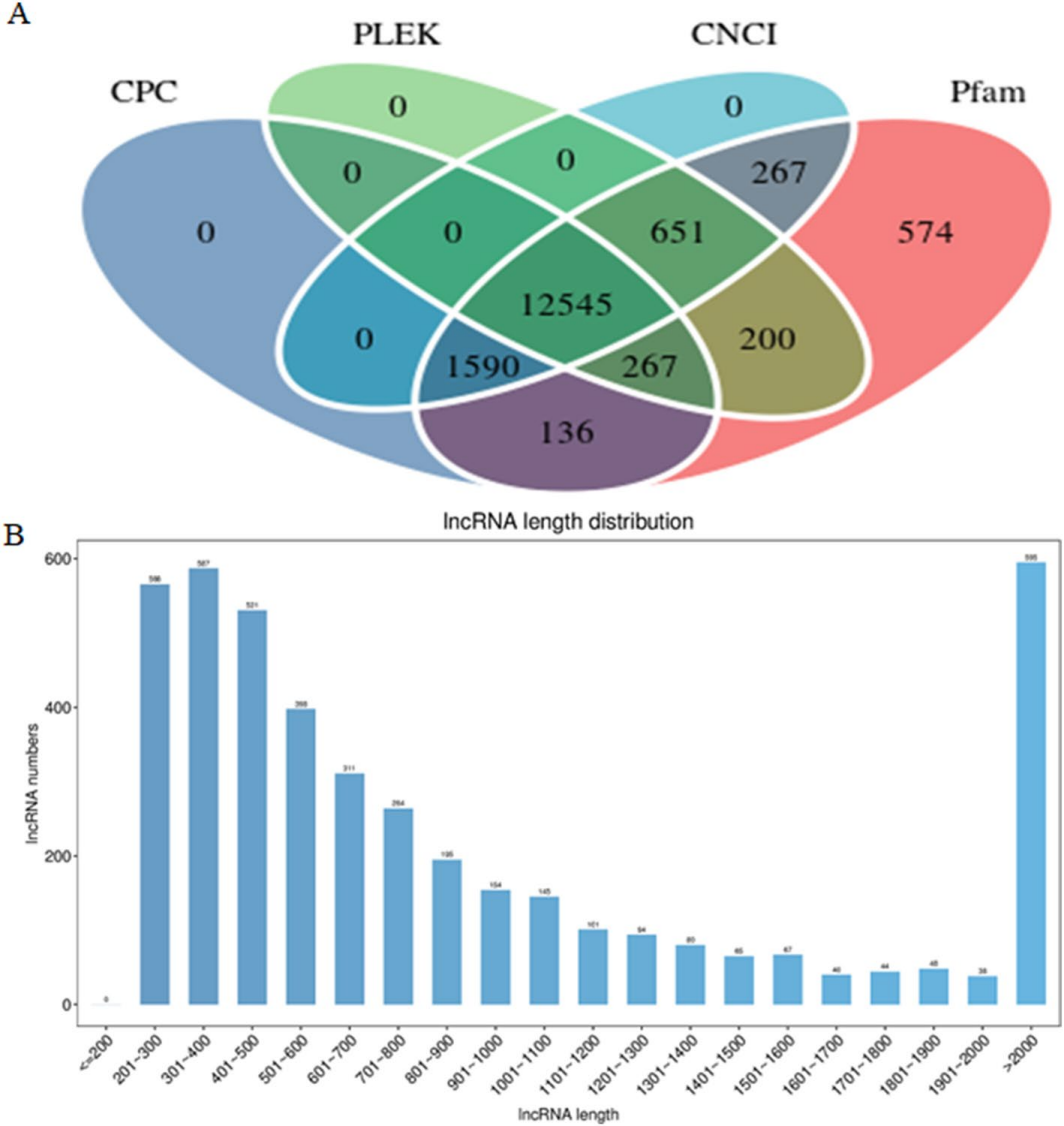
The category of lncRNAs and length distribution. (**A**) Five types of the identified lncRNAs. (**B**) The identified lncRNAs length distribution.

Long non-coding RNAs (lncRNAs) without coding activity and longer than 200 nucleotides (nt) were selected. Four screening methods were utilized, primarily CPC^14^, CNCI^15^, Pfam^16^ and PLEK^17^, to remove transcripts with coding activity. A Venn diagram showed the screening yielded 4323 potential lncRNAs with an average length of 1259.63 nt (Fig. 2A), among which 2645 lncRNAs were over 200 nt and 1318 lncRNAs were more than 1000 nt. The length of the shortest lncRNA was only 201 nt (Fig. 2B).

### Identification of differential expressed lncRNAs from PE vs. HPW

**Figure 3:**
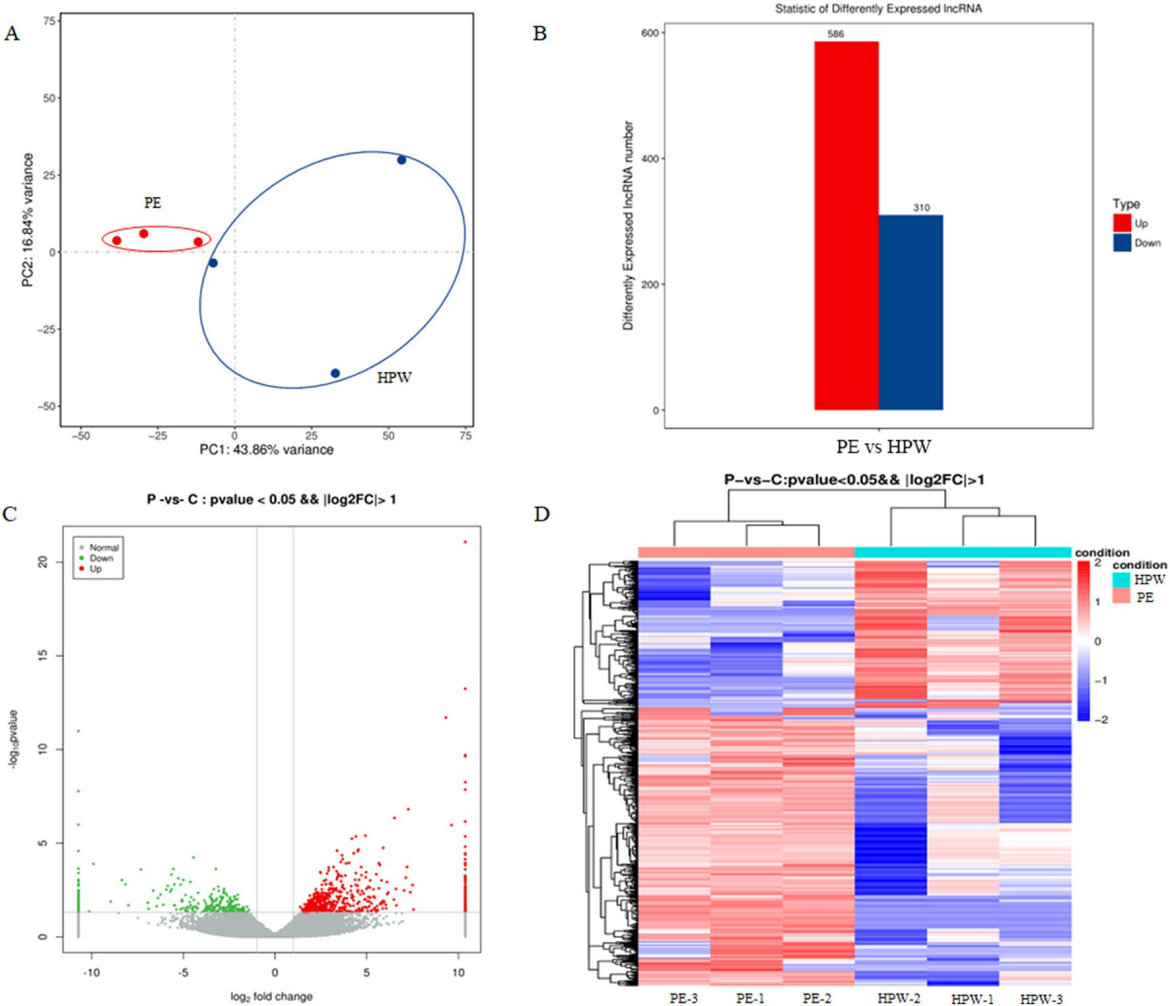
Identification of differential expressed lncRNAs from PE vs. HPW. (**A**) Principal component analysis (PCA). (**B**) The differential expressed lncRNAs were calculated by p-value ≤ 0.05 and fold change ≥ 2. (**C**) The differential expressed lncRNAs were shown in the Volcano plot. (**D**) The differential expressed lncRNAs were shown with hierarchical clustering.

Principal component analysis (PCA) was used to understand the expression pattern of lncRNAs in preeclampsia (PE) vs. HPW and showed a total of 60.7% variance (PCA1 and PCA2), indicating that the samples were relatively concentrated in similar groups (Fig. 3A). After lncRNA count normalization with DESeq^35^, two parameters—p-value < 0.05 and an absolute value of the log2FC greater than 1 were used to screen for differentially expressed lncRNAs between the preeclampsia and HPW groups. A total of 896 differentially expressed lncRNAs were identified, of which 586 were upregulated and 310 were downregulated in the preeclampsia vs. HPW groups (Fig. 3B). These differentially expressed lncRNAs were visualized in a Volcano Plot (Fig. 3C). Unsupervised hierarchical clustering was performed, which clearly distinguished upregulated and downregulated lncRNAs between the PE and HPW groups (Fig. 3D).

### GO enrichment analysis of differential expressed lncRNAs

**Figure 4:**
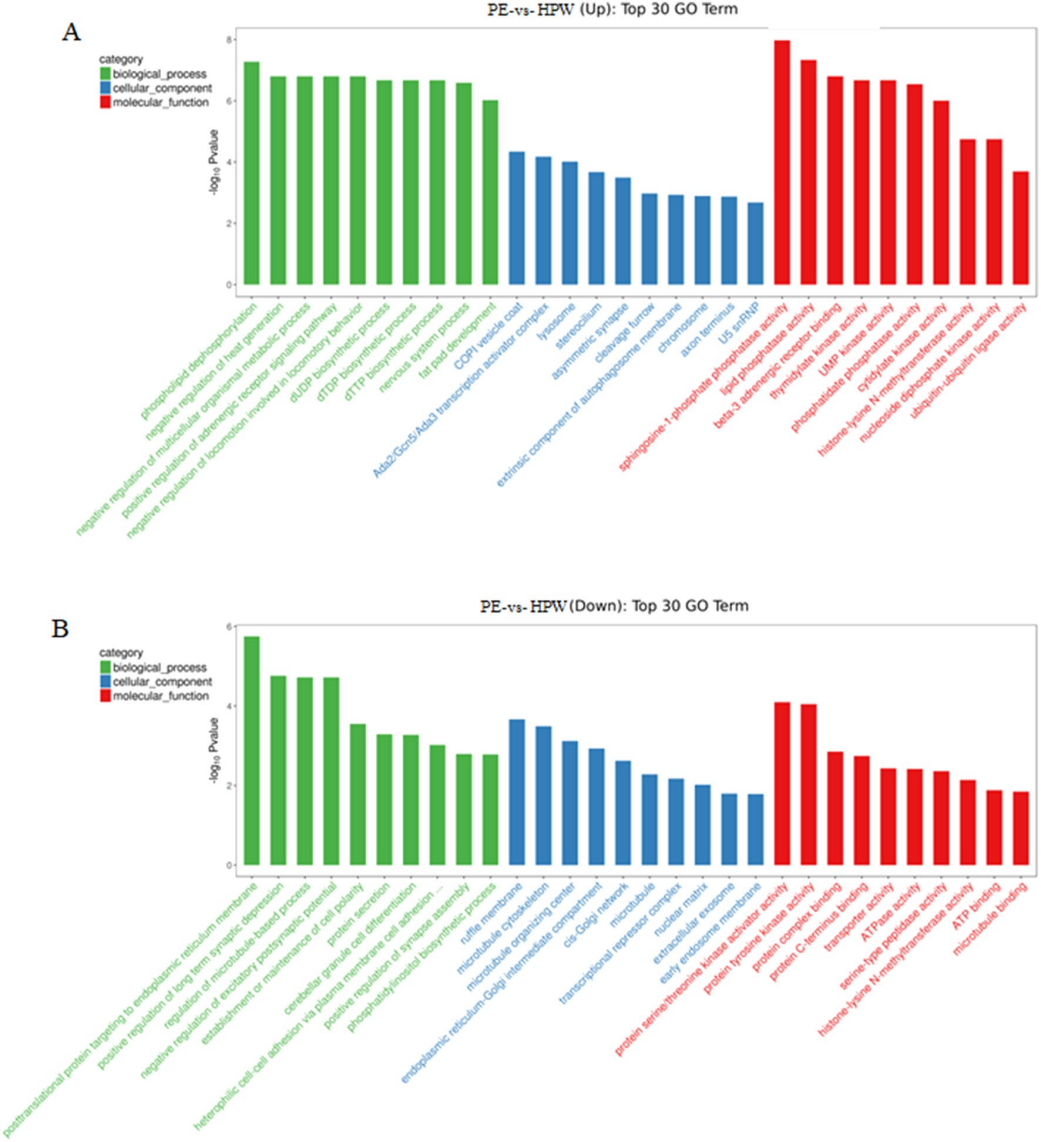
GO enrichment analysis of differential expressed lncRNAs. GO enrichment analysis was performed on differential expressed lncRNAs according to GO functional annotation information of lncRNA-derived genes. According to the GO enrichment analysis, it was mainly divided into three types including biological process, cellular component, and molecular function. (**A**) The 30 GO terms of upregulated lncRNAs with their derived genes. (**B**) The 30 GO terms of downregulated lncRNAs with their derived genes.

GO enrichment analysis divided the differential expressed lncRNAs into biological process, cellular component, and molecular function. The top 30 GO terms of the GO enrichment analysis for the upregulated lncRNAs with their derived genes were phospholipid dephosphorylation, negative regulation of heat generation, negative regulation of multicellular organismal metabolic processes, positive regulation of adrenergic receptor signaling pathways, negative regulation of locomotion involved in locomotory behavior, dUDP, dTDP, and dTTP biosynthetic processes, nervous system processes, and fat pad development in the biological process. Meanwhile, sphingosine-1-phosphate phosphatase activity, lipid phosphatase activity, beta-3 adrenergic receptor binding, thymidylate kinase activity, UMP kinase activity, phosphatidate phosphatase activity, cytidylate kinase activity, histone-lysine N-methyltransferase activity, nucleoside diphosphate kinase activity, and ubiquitin-ubiquitin ligase activity were enriched in the molecular function (Fig. 4A). The top 30 GO terms of the GO annotation analysis for the downregulated lncRNAs with their derived genes were posttranslational protein targeting to the endoplasmic reticulum membrane, positive regulation of long-term synaptic depression, regulation of microtubule-based processes, negative regulation of excitatory postsynaptic potential, establishment or maintenance of cell polarity protein secretion, cerebellar granule cell differentiation, heterophilic cell–cell adhesion via plasma membrane cell adhesion molecules, positive regulation of synapse assembly, and phosphatidylinositol biosynthetic process in the biological process. Meanwhile, protein serine/threonine kinase activator activity, protein tyrosine kinase activity, protein complex binding, protein C-terminus binding, transporter activity, ATPase activity, serine-type peptidase activity, histone-lysine N-methyltransferase activity, ATP binding, and microtubule binding were enriched in the molecular function (Fig. 4B).

### KEGG enrichment of differential expressed lncRNAs

**Figure 5:**
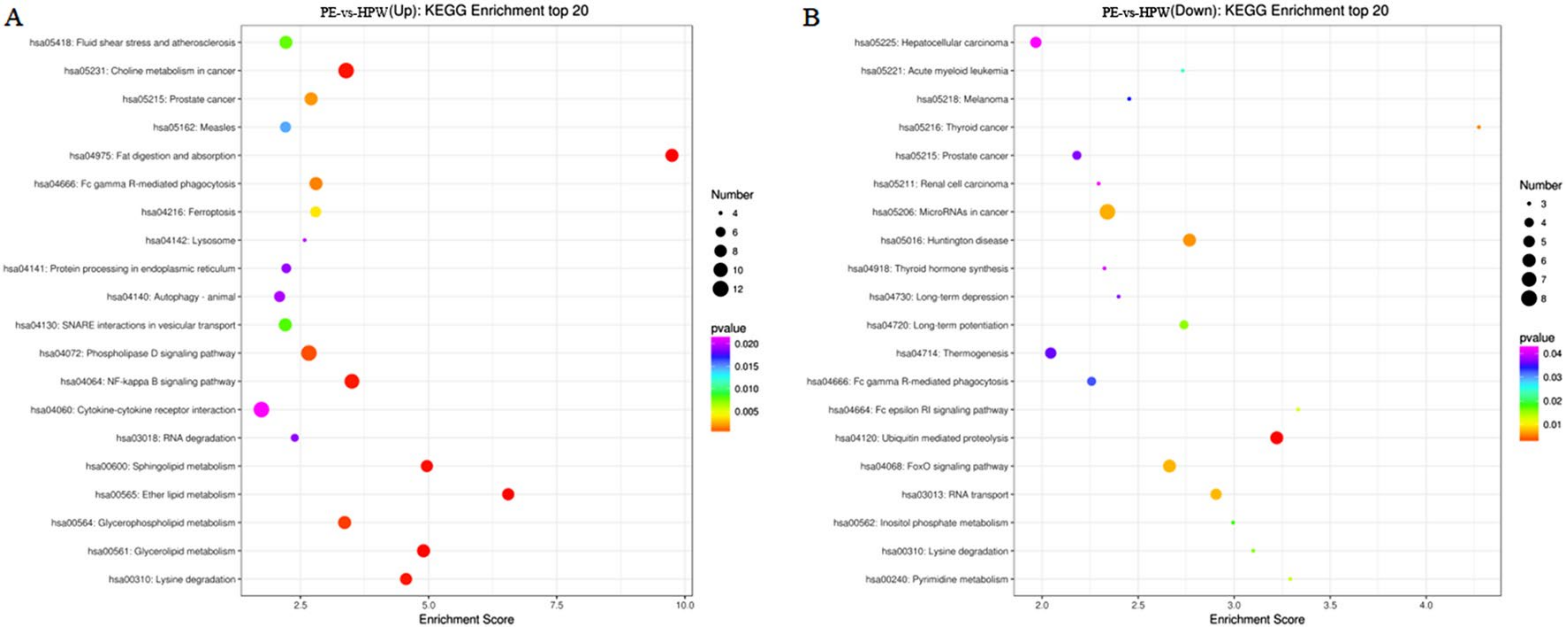
KEGG enrichment of differential expressed lncRNAs. (**A**) The KEGG analysis enriched upregulated lncRNAs in the related pathways. (**B**) The KEGG analysis enriched downregulated lncRNAs in the related pathways.

A hypergeometric distribution test was utilized to analyze the significance of differential lncRNA enrichment in each pathway entry for KEGG analysis. The upregulated lncRNAs were enriched in pathways like fat digestion and absorption, ether lipid metabolism, glycerolipid metabolism, sphingolipid metabolism, choline metabolism in cancer, lysine degradation, NF-kappa B signaling pathway, glycerophospholipid metabolism, phospholipase D signaling pathway, Fc gamma R-mediated phagocytosis, prostate cancer, ferroptosis, fluid shear stress, and atherosclerosis, SNARE interactions in vesicular transport, measles, RNA degradation, protein processing in endoplasmic reticulum, autophagy—animal, lysosome, and cytokine-cytokine receptor interaction (Fig. 5A). The downregulated lncRNAs were enriched in pathways like ubiquitin-mediated proteolysis, thyroid cancer, Huntington's disease, microRNAs in cancer, FoxO signaling pathway, RNA transport, Fc epsilon RI signaling pathway, pyrimidine metabolism, long-term potentiation, lysine degradation, inositol phosphate metabolism, acute myeloid leukemia, Fc gamma R-mediated phagocytosis, melanoma, thermogenesis, long-term depression, prostate cancer, thyroid hormone synthesis, hepatocellular carcinoma, and renal cell carcinoma (Fig. 5B).

### Construction of ceRNA networks

**Figure 6:**
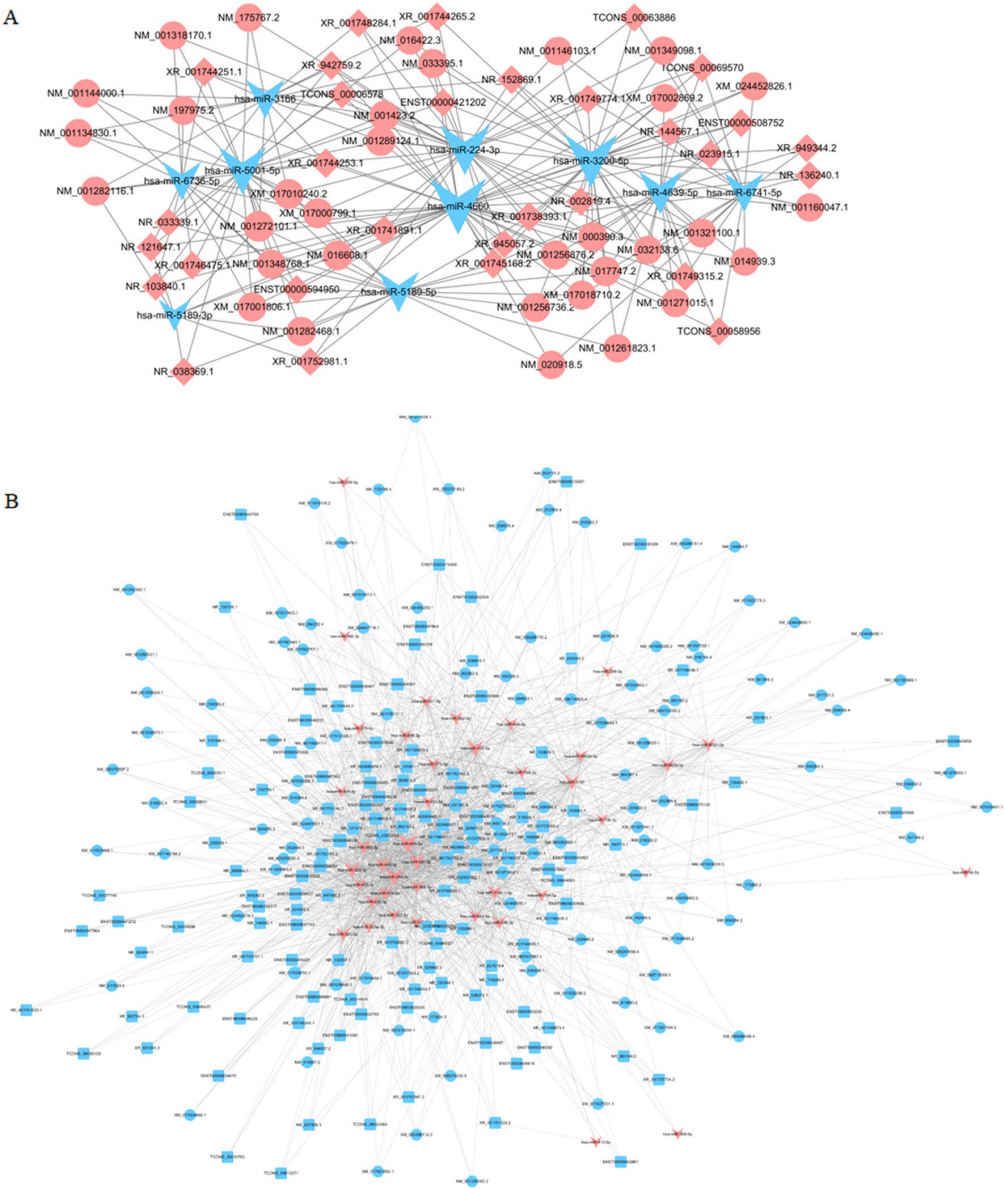
Construction of ceRNA networks. Two ceRNA interaction networks were built with ceRNA_score. (**A**) upregulated lncRNAs mediated ceRNA. (**B**) downregulated lncRNAs mediated ceRNA.

To explore the potential regulatory roles of differentially expressed lncRNAs in preeclampsia, long non-coding RNA competing endogenous RNA (lncRNA ceRNA) networks were built. Differential miRNAs and mRNAs were retrieved from the database, and the differential expressed lncRNAs were blasted with miRbase to explore the miRNA precursors, predicting 2224 pre-miRNAs. The regulatory relationship was based on lncRNA, miRNA, and mRNA, and two ceRNA interaction networks were built with ceRNA_score. One ceRNA network consisted of 31 upregulated lncRNAs, 11 downregulated miRNAs, and 34 upregulated mRNAs (Fig. 6A), while another ceRNA network contained 128 downregulated lncRNAs, 40 upregulated miRNAs, and 113 downregulated mRNAs (Fig. 6B).

## Discussions

Pre-eclampsia (PE) is a severe obstetric syndrome that has a strong association with maternal mortality^3^. Nevertheless, the underlying causes of PE remain unclear. Various studies have reported on the diverse factors that contribute to the development and progression of PE. Non-coding RNAs, specifically long non-coding RNAs (lncRNAs), have been identified to play significant roles in the development of PE. In addition, increasing data showed lncRNAs play critical roles in multiple cancers and other diseases. Nonetheless, the regulatory mechanisms of lncRNAs in PE, particularly via lncRNA-mediated competing endogenous RNA (ceRNA) networks, remain poorly understood. In this study, we comprehensively investigated the expression of lncRNAs in PE and identified that hundreds of lncRNAs were significantly differentially expressed, which were predominantly related to lipid metabolism and immunity pathways based on bioinformatics analysis.

Notably, PE can occur at any point after 20 weeks of gestation and can be classified into two subtypes: early onset PE (before 34 weeks) and late-onset PE (after 34 weeks)^36^. Previous studies indicated that poor placental development is closely related to PE. Consequently, many studies have focused on identifying the regulatory mechanisms of lncRNAs in placental tissue in PE and have predicted their potential as biomarkers of PE or for their involvement in the pathogenesis of PE through lncRNA-mediated ceRNA networks^19–25^.

However, analyzing the expression of lncRNAs in whole blood samples from PE patients, while excluding red blood cells, may be more relevant and useful for the development of biomarkers. Furthermore, studies have suggested that metabolic abnormalities of steroid hormones in placental tissue may have a significant role in the etiology of PE^12^. Therefore, we investigated the distinct expression profiling of lncRNAs in the whole blood removing the red blood cell may be more meaningful. Our analysis identified 896 significantly differentially expressed lncRNAs between PE and healthy pregnant women, comprising 586 upregulated and 310 downregulated lncRNAs. We also conducted gene ontology and Kyoto Encyclopedia of Genes and Genomes pathway analyses to gain a deeper understanding of the regulatory roles of these lncRNAs and their associated genes. Our analysis indicated that the upregulated lncRNAs primarily influenced pathways related to the metabolism of fats, such as fat digestion and absorption, ether lipid metabolism, glycerolipid metabolism, and sphingolipid metabolism. They were also associated with lysine degradation, the NF-kappa B signaling pathway, glycerophospholipid metabolism, phospholipase D signaling pathway, choline metabolism in cancer, and Fc gamma R-mediated phagocytosis. The downregulated lncRNAs, on the other hand, were primarily associated with ubiquitin-mediated proteolysis, thyroid cancer, Huntington's disease, microRNAs in cancer, FoxO signaling pathway, RNA transport, Fc epsilon RI signaling pathway, pyrimidine metabolism, long-term potentiation, and lysine degradation.

Furthermore, we investigated the regulatory mechanisms of these lncRNAs via ceRNA analysis. ceRNA regulatory mechanism suggests that some RNAs including lncRNAs contain miRNA response element, which sites competitively bind miRNAs and subsequently impact their target genes’ expression. The role of ceRNA regulatory networks has been extensively studied in various diseases. Besides, lncRNA PGK1P2 was confirmed to regulate the expression level of Phosphoglycerate kinase 1 via miR-330-5p in PE^25^. An identified ceRNA network in early onset PE consisted of 21 lncRNAs, 3 mRNAs, and 69 miRNAs and could potentially be involved in the regulatory process of PE^23^. According to one study, Lnc-CTD-2383M3.1 may regulate ADAMTS6 expression via miR-210-3p sponging and may potentially have a regulatory role in the development of PE^22^. However, the etiopathogenesis of PE is still vague. To gain further insight into the etiopathogenesis of PE, we constructed two ceRNA regulatory networks using the ceRNA_score algorithm, with one network consisting of 31 lncRNAs, 11 miRNAs, and 34 mRNAs, and the other one containing 128 lncRNAs, 40 miRNAs, and 113 mRNAs.

Additionally, the ceRNA networks involved several hub genes, including ephrin B1, pannexin 1 (Panx 1), a member of the solute carrier family, IL-6, and TNF receptor superfamily members^37^. Panx 1 and TLR4 might induce ferroptosis in PE via solute carrier family 7 member 11-mediated signaling pathways^38^. The IL-6/STAT3 signaling pathway could be repressed by pravastatin to alleviate oxidative stress, improve preeclampsia, and decrease the apoptosis of placental trophoblastic cells in rats with PE^39^. In pregnancy, Tumor necrosis factor-alpha (TNF-α) was closely associated with hormone synthesis, placental architecture, and embryonic development, and the increasing levels of TNF-α were related to pregnancy loss and PE^40^. Epidermal Growth Factor-like Domain 7 mediated trophoblast migration and invasion and plays a role in placental development^41^. Matrix metalloproteinases-2 and -9 might be developed as biomarkers for predicting the development of PE in the first trimester^42^. circRNA zinc finger DHHC-type palmitoyltransferase 20 mediated the expression of GRHL2 via miR-144 sponges, resulting in the inhibition of proliferation, migration, and invasion in trophoblast cells^43^. These findings indicate that these target genes may be critical in the etiopathogenesis of PE, but further validation is necessary. In this study, we demonstrated that the differentially expressed lncRNAs between PE and healthy pregnant women were primarily related to pathways involving lipid metabolism, innate immunity, and ubiquitin-mediated proteolysis. In addition, a large subset of target genes was mediated by the lncRNA-mediated ceRNA networks and the rols of these dysregulated lncRNAs were still need further validated in the etiopathogenesis of PE. Taken together, these findings provide new insights into the underlying mechanisms of PE.

## Data Availability

The RNA sequencing data were deposited into the published data with the accession number of PRJNA665923 and are available at the following URL: https://www.ncbi.nlm.nih.gov/sra/?term=PRJNA665923. mRNA Database: ftp://ftp.ncbi.nlm.nih.gov/genomes/all/GCF/000/001/405/GCF_000001405.38_GRCh38.p12/GCF_000001405.38_GRCh38.p12_rna.fna.gz
